# A Systematic Review of Aircraft Disinsection Safety, Toxicity, and Tolerability

**DOI:** 10.3390/toxics13110965

**Published:** 2025-11-09

**Authors:** Michael Klowak, Gregory D. Hawley, Syed Zain Ahmad, Candice Madakadze, Aquilla Reid-John, Jahmar Hewitt, Asal Adawi, Andrea K. Boggild

**Affiliations:** 1 Tropical Disease Unit, Toronto General Hospital, University Health Network, Toronto, ON M5G 2C4, Canada; 2 Institute of Medical Science, University of Toronto, Toronto, ON M5S 3H2, Canada; 3 Department of Medicine, University of Toronto, Toronto, ON M5S 3H2, Canada; 4 Department of Physiology, University of Toronto, Toronto, ON M5S 3K3, Canada; 5 Temerty Faculty of Medicine, University of Toronto, Toronto, ON M5S 3K3, Canada

**Keywords:** aircraft, disinsection, insecticide, marine vessel, occupational health, pesticide, safety, tolerability, toxicity

## Abstract

Treatment of aircraft with insecticide in a procedure referred to as ‘disinsection’ is recommended to prevent the conveyance of arthropod vectors internationally and to mitigate the globalization of vector-borne infectious diseases. However, the full spectrum of human-based outcomes related to disinsection of conveyances has not been recently synthesized. A systematic review was conducted to evaluate the human safety and toxicity of insecticides used during the process of disinsecting international aircraft, marine vessels, rail, and ground transportation of mosquitoes. The systematic review was conducted according to the PRISMA guidelines and was registered in PROSPERO (CRD42024543998). The certainty of the evidence was rated, and key primary outcomes, including human health effects of conveyance disinsection, were synthesized. A total of 21 studies that described human health effects of conveyance disinsection and reported outcomes of safety, toxicity, and tolerability were included, and were of generally limited quality and high risk of bias, with low to very low certainty of estimates of effect. No high-quality studies investigating the safety, toxicity, or tolerability of disinsection were identified. Human health effects, including morbidity, including work days lost, adverse events including hospitalization, objective measures of insecticide toxicity, detectable and elevated urinary metabolites, and subjective reporting of symptoms consistent with acute insecticide poisoning, were reported by the small number of uncontrolled observational studies and public health surveillance reports included. Given the reports of significant morbidity, adverse events, and toxicity putatively attributable to aircraft disinsection, well-designed studies in exposed populations investigating the full range of human health impacts of disinsection on passengers and crew are urgently needed.

## 1. Introduction

Aircraft ‘disinsection’ is a “procedure whereby health measures are taken to control or kill the insect vectors of human disease present in baggage, cargo, containers, conveyances, goods and postal parcels (International Health Regulations [IHR], Part I, article 1)” [1] to prevent international dispersal of mosquitoes and the diseases they vector. In practice, this process necessitates the application of aerosolized or liquid insecticides such a permethrin or d-phenothrin by ground crew and/or flight attendants to cabin surfaces and within cabin air. According to the IHR, disinsection should “be carried out so as to avoid injury and, as far as possible, discomfort to persons” (IHR, Part IV, article 22, Section 3) [1]; however, large-scale syntheses of human studies demonstrating the safety of disinsection procedures in highly exposed individuals, such as flight attendants and ground crew, are absent from the medical literature.

The first edition of the World Health Organization’s (WHO) comprehensive publication on aircraft disinsection methods and procedures, building on ad hoc earlier publications over the past several decades, was published in 2021 [2] and then updated in 2023 [3]. The updated document provides newer guidance on how to apply insecticides and the equipment used therein (Supplementary Table S1), type of spray specifications (i.e., aerosol vs. residual), updated tables for calculating amounts of aerosol spray required, newer protocols for pre-boarding and pre-departure cabin treatment, and the International Civil Aviation Organization (ICAO) certification requirements for the disinsection procedures. Many countries have developed specific aircraft disinsection regulations, which can be found in Supplementary Table S2. Moreover, marine conveyance disinsection regulations also exist, and countries requiring such procedures aboard marine vessels can be found in Supplementary Table S3.

Vehicles, including marine vessels, rail and ground transportation, and aircraft, contribute to the globalization of vector-borne infections, including arboviruses (e.g., dengue, chikungunya, Zika) and malaria through the conveyance of infected persons along with the mosquito and other vectors capable of pathogen transmission. Consequently, conveyance disinsection through the use of insecticides, including aerosol sprays, has been used to eliminate arthropod vectors of public health importance, including mosquitoes. However, despite the widespread use of disinsection, comprehensive guidance documents regarding the safety and toxicity of such procedures to human health are largely unavailable. As such, we undertook a systematic review to synthesize the literature around the human health effects of conveyance disinsection and report our findings herein. We also identify areas warranting future concerted research efforts.

## 2. Methods

A systematic review of conveyance disinsection was commissioned by the WHO [4] in accordance with the PICO framework below (Table 1). As a main outcome beyond effectiveness, the systematic review synthesizes the safety, toxicity and tolerability of mosquito disinsection of any areas of international conveyances, including the passenger chambers, cargo areas, and cargoes, of aircraft, marine vessels, and rail and ground transport vehicles, to prevent or reduce the spread of competent vectors of human disease via international travel (Table 1).

The systematic review was conducted according to the Preferred Reporting Items for Systematic Reviews and Meta-Analysis (PRISMA) guidelines [6] and registered with the International Prospective Register of Systematic Reviews (PROSPERO) database (CRD42024543998).

**Inclusion criteria**: All papers reporting on mosquito disinsection of any areas of any international conveyance, including aircraft, marine vessels, ground, and rail transport, according to the insecticide used (including but not limited to the most commonly used and historical insecticides such as DDT, d-phenothrin, and permethrin), and method of application were included. All of the following methodologies were included: systematic reviews, randomized controlled trials (RCTs), cohort studies, cross-sectional studies, case–control studies, case-series, and case reports (n ≥ 1). Studies with alternative methodological designs (e.g., conference abstracts) were also included, so long as they reported primary data.

**Exclusion criteria**: We excluded in vitro and animal studies as well as those that did not allow ascertainment and/or assessment of our pre-defined safety, toxicity, and tolerability outcomes (e.g., mathematical modeling studies). Studies that were carried out in models replicating conveyance interiors but which did not fully reproduce the conveyance environment (e.g., non-pressurized structure instead of an actual aircraft cabin; upholstery sections impregnated with insecticide in a laboratory rather than actual residual disinsection of an aircraft, etc.).

**Outcomes**: The PICO statement above describes the outcomes of relevance to the safety, toxicity, and tolerability systematic review (Table 1). The primary outcome addressed in the systematic review and from which the Summary of Findings tables were generated includes disinsection safety and toxicity, with proportionate and absolute human adverse events, objective measures of biological toxicity, levels of pyrethroids and their metabolites in human tissues and effluents, and objective and subjective measures of tolerability, morbidity, and mortality as the main outcomes of interest.

**Search strategy**: Electronic databases including PubMed, Embase, Medline, Scopus, LILACS, and CINAHL were searched from inception to 31 May 2025 without language restriction as previously described [4] using combinations of search terms related to disinsection, insecticides (e.g., DDT, permethrin, etc.), types of conveyance (e.g., airplane, ship, train, lorry, etc.), transportation sector (e.g., aviation, shipping, rail, ground transportation), relevant occupational hazards (e.g., flight attendant, crew, etc.), mosquitoes, and relevant vector-borne diseases disseminated via international conveyances (e.g., malaria, dengue, Zika, etc.). The exact search terms employed, as previously reported [4] were: (disinsection OR insecticide OR d-phenothrin OR permethrin OR deet OR spraying OR “mosquito control”) AND (travel OR airport OR airplane OR plane OR aviation OR aircraft OR airline OR air-cans OR truck OR bus OR cargo OR rail OR train OR tram OR marine OR ship OR boat OR lorry OR vessel OR submarine OR space OR spacecraft OR rocketship OR spaceship OR “marine vehicle” OR “marine vessel” OR “cruise ship” OR “water taxi” OR ferry OR barge OR “passenger chamber” OR “cargo area” OR “land transport vehicles”) AND (neurotoxicity OR crew OR passengers OR “flight attendant” OR “occupational exposure” OR insect OR mosquito OR malaria OR “airport malaria” OR dengue OR chikungunya OR zika).

Literature not appearing in the six targeted databases, including conference proceedings, dissertations, and other documents existing in the public domain, was captured by searching databases such as OpenGrey and the Grey Literature Report. A hand search of the bibliographies of key papers was executed to capture applicable literature not otherwise captured. The online platform Covidence was used for overall study management, deduplication, title, abstract, and full-text screening. Articles were double-screened independently by two reviewers, with discrepancies resolved via discussion and/or by a third arbitrator.

Two independent reviewers conducted the data extraction, which was validated by the study lead according to the Grading of Recommendations, Assessment, Development, and Evaluation (GRADE) framework [7,8,9]. Articles not written in English were screened and extracted by reviewers speaking the relevant language (e.g., French, Spanish) or were translated into English using Google Translate (Google, Mountain View, CA, USA). Non-English language full-texts sent to the study team by the University of Toronto’s interlibrary loan system as image files were saved as PDF files and then processed through optical character recognition software [10,11,12] in order to facilitate Google translation. Any discrepancies that arose were resolved by conversation between reviewers, and any disagreements were adjudicated by a tertiary author. Following data extraction, standard “study characteristics” tables were created, and then data were synthesized in aggregate both quantitatively and qualitatively, where applicable. Standard “Summary of Findings” tables were then generated using GRADEpro GDT (Cochrane, Oxford, UK and McMaster University, Hamilton, ON, Canada, 2025). If outcomes were reported inconsistently or when different types of data were collected and reported across studies, preventing numerical synthesis, narrative synthesis was conducted and represented in text format only.

**Data Analysis**: Sample sizes, means with standard deviations, mean differences, and/or medians with ranges and interquartile ranges were reported for continuous variables. Frequencies and proportions with 95% confidence intervals were reported for dichotomous or categorical variables (such as the presence of adverse event, for example) when provided. We planned to collect continuous outcomes (mean difference), and dichotomous outcomes (relative risk, and odds ratio) when available, and reported them in Summary of Findings tables if and when the study producing primary data included a comparator group, using a standardized measure of treatment difference. Summary estimates of both continuous and dichotomous outcomes were pooled for each combination of disinsection exposure (including insecticide used and application method) and safety outcome (e.g., biological toxicity, objective morbidity, subjective tolerability, etc.). The level of significance was set at a 5% alpha level for summary estimates of outcomes measured against a comparator. Statistical analyses were executed using GRADEpro GDT (Cochrane, Oxford, UK and McMaster University, Hamilton, ON, Canada, 2025), GraphPad PRISM v.9.0 (GraphPad, La Jolla, CA, USA), and Review Manager (RevMan, computer program, version 5.3. Copenhagen: The Nordic Cochrane Centre, The Cochrane Collaboration, 2014).

Included studies were examined for key health equity factors represented by the acronym PROGRESS, which includes place of residence; race or ethnicity; occupation; gender and sex; religion; education; socioeconomic status; social capital or resources; and additional relevant stratifiers such as sexual orientation, marital status, and gestational status, as previously reported [13]. We also included other health equity and human rights stratifiers signified by the acronym CANDALS—citizenship; ability; neurotypicality or neurodiversity; disability; age; literacy and/or fluency in a universal language of aviation; and size, body mass index (BMI) or body habitus, as described previously [14]. Studies were reviewed for any description of and/or data stratification by the PROGRESS-CANDALS factors.

**Risk of Bias and Certainty of Evidence**: Forms to score risk of bias (ROB) were adapted in accordance with standard critical appraisal tools developed by the Joanna Briggs Institute, were designed, and populated independently and simultaneously by two reviewers to execute the bias assessment [15]. Ascertainment of methodological quality followed the GRADE framework by scoring each included study a ‘quality grade’ of high, moderate, low, or very low, based on the ascertained level of bias [7,8,9]. Discussion resolved discrepancies, and in the case of non-agreement, a tertiary arbitrator was engaged. Outcomes either inadequately or not fully reported, or assessments obtained only subjectively (such as by self-report of adverse event, for example) meant that risk for reporting and/or information/outcome bias, respectively, were present. Assessments of bias were aggregated, and an overall ROB score was ascertained for each study. The aggregate bias risk was rendered according to the degree (i.e., adequate or not) of allocation, concealment, blinding, attrition, and completeness of reporting, where noted and applicable to the study design, by one reviewer and then validated by a second reviewer. The software RevMan (version 5.3), Copenhagen: The Nordic Cochrane Centre, The Cochrane Collaboration, 2014, was used to generate heatmaps of study quality.

Further GRADE measures such as outcome inconsistency, indirectness, and imprecision, along with degree of publication bias and likely confounding, effect size, and relevant dose–response gradients were noted, where applicable, when evaluating certainty of evidence. Overall, ROB was considered in conjunction with these additional GRADE measures to generate one final, consolidated certainty of evidence GRADE score, according to each reported safety, toxicity, and tolerability outcome, whether objective or subjective in nature.

Sources of expected effect heterogeneity likely to affect the safety outcomes included but were not limited to: specifications of the insecticide applied (including type, formulation and concentration, such as 2% permethrin vs. 2% d-phenothrin, for example); whether or not the air filtration system of the conveyance was operational during application of insecticide and, if so, the number of air exchanges per unit time on the conveyance; model of the particular conveyance having insecticide applied (e.g., specific models of aircraft, type of marine vessel, etc.); climate-related measures including ambient temperature, humidity, UV index, cabin pressure, and altitude during insecticide application; and the precise time of travel during which insecticide application occurred (e.g., at pre-embarkation vs. taxiing vs. time of descent). The disinsection *process* constitutes a multistep sequence with variability inherent to individuals applying insecticide, and is, therefore, a “complex intervention”. Individuals of diverse height, strength, and stride cadence are expected to apply insecticides nonuniformly and, therefore, generate potential effect heterogeneity. The extent and rate of insecticide dispersion are most likely influenced by climatic and ambient conveyance factors, including but not limited to operation of the air conditioner, as is recommended by authoritative guidance [16]. Each of these types of factors was considered and extracted in as granular a form as was reported by the primary study and subsequently noted in the Study Characteristics tables. A lack of such granularity was indicated in the limitations of data generalizability and external validity section of the descriptive text.

## 3. Results

### 3.1. Literature Search

**Figure 1:**
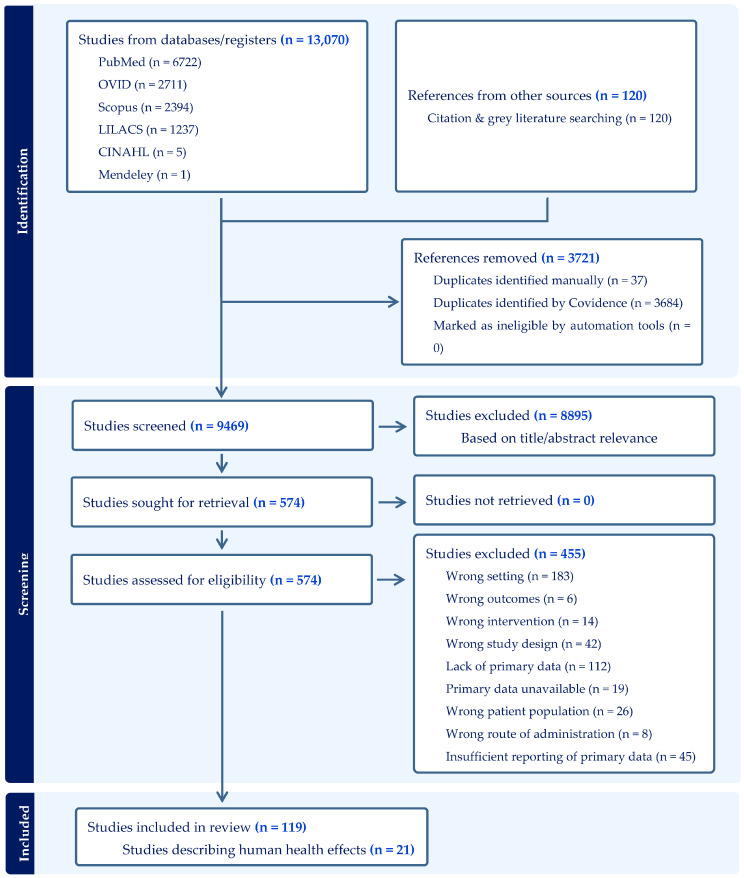
PRISMA flow diagram of literature examined for inclusion in the systematic review of safety, toxicity, and tolerability of international conveyance mosquito disinsection.

Of the 9469 unique articles identified in the search, 574 moved through to full-text screening, after which 455 were excluded for not fulfilling inclusion criteria (Figure 1). This review forms part of a broader systematic review series evaluating the disinsection of vehicular conveyances across multiple domains, including efficacy, human health effects, and epidemiological outcomes. Of 119 unique included articles or studies identified across the entire systematic review series, 21 reported primary human health effects, including toxicity, subjective tolerability, morbidity, and/or mortality. These publications are the focus of this specific systematic review and will be exclusively discussed herein.

### 3.2. Included Studies

Table 2 pertains to studies reporting the safety, toxicity, and tolerability of aircraft disinsection in humans with primary reported outcomes of morbidity, adverse events, objective measures of toxicity, and subjective measures of tolerability.

#### Safety and Toxicity of Disinsection

A total of 21 studies evaluating the human safety, toxicity, and/or tolerability of aircraft mosquito disinsection fulfilled inclusion criteria (Table 2), of which 11 were experimental trials without human control arms [17,18,19,20,21,22,23,24,25,26,27], 1 was a case–control study with a comparator arm of unexposed Arizona residents [28], 1 was a cohort study with both exposed and unexposed flight attendants [29], 5 were case series [30,31,32,33,34], 1 was a case report [35], and 2 were review articles reporting primary data [36,37].

Of 21 studies evaluating or reporting on human safety, toxicity, and tolerability of aircraft disinsection of mosquitoes, 3 studies reported significant chronic morbidity [28,32,34], 3 studies reported specific adverse events and health safety effects [32,34,35], 12 studies reported objective signs and/or biological markers of exposure and/or toxicity [17,25,26,27,28,29,30,31,37], 11 studies reported on subjective symptoms in those exposed [18,20,26,28,30,32,33,34,35,36,37], and 8 studies reported on subjective tolerability with or without subjective symptoms [19,20,21,23,24,34,36,37].

### 3.3. Summary of Methodological Quality and Risk of Bias Assessment

**Figure 2:**
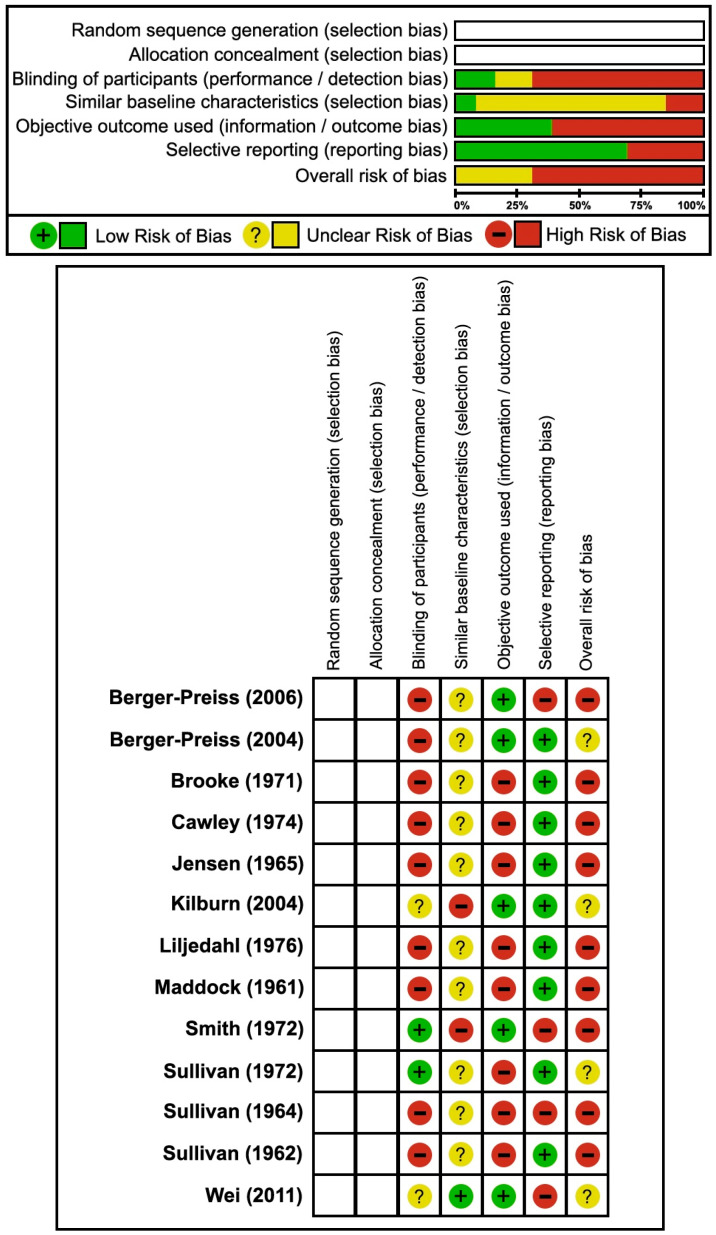
Risk of bias assessment for studies reporting the safety, toxicity, and tolerability of conveyance disinsection. Studies cited: [17,18,19,20,21,22,23,24,25,26,27,28,29].

Of the 21 studies included in this review, only 13 provided sufficient methodological information to permit structured risk-of-bias assessment, as the remaining eight were case reports or case series that lacked the data necessary for domain-level evaluation. Overall, aircraft disinsection studies reporting human safety, toxicity, and tolerability were of low quality and high risk of bias (Figure 2, Table 3), with only two human studies reporting an unexposed human comparator or control arm [28,29]. Only one study [29] of aircraft disinsection evaluating safety or toxicity in humans adhered to standard study design or reporting of methodological rigor including: approval by institutional review boards or research ethics committees governing human subjects considerations in research; description or notation of recruitment process, eligibility assessment, and informed consent of participants; study protocol registration; a statement of independence of investigators from stakeholders and disclosures of funding; and collection and reporting of objective biological, behavioral, or psychological metrics corroborating subjectively reported tolerability, and more notably, intolerability. Despite this, although all included studies implemented disinsection as an intervention, none incorporated randomization or allocation concealment procedures consistent with formal interventional trial methodology. Therefore, two domains under “Selection Bias” in Figure 2 were intentionally left blank to illustrate this absence. Collectively, studies of human safety, toxicity, and/or tolerability of aircraft mosquito disinsection were either at serious risk of bias for morbidity outcomes [28,32,34] or very serious risk of bias for safety, toxicity, and tolerability outcomes [19,26,27,28,30,31,32,33,34,35], with estimated health effects of very low certainty according to GRADE assessment.

**Table 2:** Characteristics of studies examining the safety and toxicity conveyance disinsection that were included in the systematic review.

Author (Year)	Study Design	Country Setting	Conveyance	Population	Sample Sizes	Mean Age (SD)	Range	Sex N (F:M)	Insecticide Used	Formulation	Disinsection Method
Berger-Priess (2006) [17]	Experimental Trial	Germany	Grounded passenger aircraft (Airbus A310 and Boeing 747-400)	Study personnel	4–6	ND	ND	ND	D-phenothrin	D-phenothrin 2%	Simulated pre-flight and top-of-descent spraying
Berger-Priess (2004) [25]	Experimental Trial	Germany	Passenger aircraft (Airbus A310)	Study personnel	4–6	ND	ND	ND	Pyrethrum extract(s), Pyrethrins	1.25% pyrethrum extract (containing 25% pyrethrins, active ingredients), synergist piperonyl butoxide (2.6%), and the propellants butane and propane	Simulated in-flight spraying method in grounded aircraft
Bitelli (1969) [36]	Review	Italy	Passenger aircraft	Passengers and crew	ND	ND	ND	ND	DDT	ND	ND
Bonta (2003) [30]	Case Series	USA	Passenger aircraft (Boeing 747-400)	Flight attendants, passengers, and pilots	38	ND	ND	ND	Permethrin	ND	Residual treatment
Brooke (1971) [18]	Experimental Trial	UK	Grounded passenger aircraft (De Havilland Comet 4C)	Authors and engineers	6	ND	ND	ND	Bioresmethrin, Resmethrin, Pyrethrins, DDT, Bioalletrhin, Tropital	Bioresmethrin: 0.05%, 0.075%, 0.1%, 0.25%; Resmethrin: 0.1%, 0.25%, 0.5%; Pyrethrins 0.4% + DDT 3.0%; Pyrethrins 0.45% + Tropital 2.7%	Simulated Blocks-away without passengers present
De Tavel (1967) [37]	Review	Switzerland	ND	Volunteers	ND	ND	ND	ND	Dichlorvos	ND	ND
Edmundson (1970) [31]	Case Series	US	Commercial aircraft	Aircraft disinsection technicians	4	49	37–60	0:4	Pyrethrins, DDT	Aerosol containing 3% DDT and 1% pyrethrin	Not specified
Kilburn (2004) [28]	Case–Control	US	Passenger aircraft	Flight attendants	E: 33 NE: 202	E: 47.7 (6.9) NE: 45 (21.1)	E: 32–60 NE: ND	ND	Pyrethroids	Not specified	Residual treatment
Liljedahl (1976) [20]	Experimental Trial	US	Commercial passenger aircraft (Boeing 707, Boeing 727)	Authors and crew	At least 18	ND	ND	ND	D-phenothrin	2% (+)-phenothrin in a 3:17 ratio of Freon-11 to 12; and in a 1:1 mixture of Freon-11 to 12	Blocks-away
Maddock (1961) [26]	Experimental Trial	US	Commercial aircraft	Study personnel	4	ND	ND	ND	Dichlorvos	Not specified	Simulated in-flight spraying method in grounded aircraft
Przyborowski (1962) [32]	Case Series	Poland	Ship	Crew members	20	ND	ND	ND	Dieldrin	Liquid preparation stored in tins and wooden crates	Contaminated food stores
Smith (1972) [27]	Experimental Trial	US	Simulated aircraft (altitude chamber)	Staff volunteers and paid participants	8	ND	21–40	2:6	Dichlorvos	Not specified, but product was 5–10× higher than median value typically prescribed for disinsection	Top-of-descent (8000 ft simulation)
Sutton (2007) [33]	Case Series	US	Commercial aircraft	Flight attendants	12	ND	ND	ND	Permethrin	Permethrin 2.2% (25:75 cis:trans)	Residual treatment
Vanden Driessche (2010) [35]	Case Report	Netherlands	Passenger aircraft	Passenger	1	29	29	1:0	D-Phenothrin	D-phenothrin, tetrafluoroetane, C11-15-iso-alkanes, methoxypropoxypropanol, peach perfume	Blocks-away
Wei (2012) [29]	Cohort	US	Commercial aircraft	Flight attendants	11 exp. 17 unexp.	ND	18–65	ND	Permethrin	Not specified	Residual treatment
Woodyard (2001) [34]	Case Series/News Report	US	Passenger aircraft	Passengers, flight attendants, and pilots	9	ND	ND	5:4	Permethrin, in one case only	Not specified	Residual treatment
‡ Cawley (1974) [19]	Experimental Trial	US	Commercial passenger aircraft (Boeing 707, Boeing 727)	Crew members	ND	ND	ND	ND	Bioresmethrin, Resmethrin, S-2539 Forte	Bioresmethrin: 2% with 5% ethanol; Resmethrin: 0.3%, 1.2%, and 2% with 5% ethanol; S-2539 Forte: 0.3%, 1.2%, and 2%	Blocks-away
‡ Jensen (1965) [24]	Experimental Trial	US	Commercial passenger aircraft (DC-6B)	Passengers and crew	28–45 per 6 flights	ND	ND	ND	Dichlorvos vapour	Air concentration ranged from 0.13 to 0.25 μg/L dichlorvos	Disinsection anytime while aircraft is closed, and ventilation system is on
‡ Sullivan (1972) [23]	Experimental Trial	US (WHO)	Commercial jet passenger aircraft (B-747, B-707, BAC 111, CD-8, DC-9)	Passengers	591 int. 68 con.	ND	ND	ND	Bioresmethrin, G-1707, Resmethrin, Pyrethrum extract(s), Tropital, (+)-trans-allethrin	Resmethrin: 1.12%, and 2.25% aerosols; Bioresmethrin: 1%, and 2% aerosols; (+)-trans-allethrin: 1.11%, and 2.22% aerosols; G-1707: pyrethrum extract (20% pyrethrins) 2.25%, Tropital synergist 2.70%, petroleum distillate 10.05%, Freon-12 59.50%, Freon-11 25.50%	Blocks-away
‡ Sullivan (1964) [22]	Experimental Trial	Fiji, New Zealand, Philippines	Passenger aircraft (DC-3, DC-7C, DC-8, Fokker, Viscount)	Passengers and flight attendants	ND	ND	ND	ND	DDT, G-1492, Pyrethrum extract(s), SRA	SRA: 1.60% pyrethrum extract (25% pyrethrins), 3.00% DDT, 7.50% Xylene, 2.90% odourless petroleum distillate, 42.50% Freon-12, 42.50% Freon-11; 6.00% G-1492: pyrethrum extract (20% pyrethrins), 2.00% DDT, 8.00% Xylene, 58.80% Freon-12, 25.20% Freon-11	Blocks-away
‡ Sullivan (1962) [21]	Experimental Trial	Italy, Switzerland, UK, US	Passenger aircraft (Boeing 707, Caravelle, Comet 4B, DC-6B, DC-8, Viscount)	Passengers and crew	ND	ND	ND	ND	DDT, G-1480, Pyrethrum extract(s), SRA	SRA: 1.60% pyrethrum extract (25% pyrethrins), 3.00% DDT, 7.50% Xylene, 2.90% odourless petroleum distillate, 42.50% Freon-12, 42.50% Freon-11; G-1480: 3.40% pyrethrum extract (20% pyrethrins), 1.17% DDT, 4.50% aromatic petroleum derivative solvents, 63.62% Freon-12, 27.31% Freon-11	Blocks-away
**Author (Year)**		**Morbidity, Adverse Events, Objective Signs of Toxicity, and Subjective Symptoms**									
Berger-Priess (2006) [17]		**Objective signs of toxicity:** The pre-embarkation method resulted in lower dermal exposures, while top-of-descent spraying resulted in lower inhalation exposures for both sprayers and passengers. However, during the pre-embarkation method of spraying, exposure is reduced to 0.1–0.5% of that of top-of-descent, for persons boarding 20 min following termination of disinsection. Urine metabolites of d-phenothrin were detected at concentrations of 0.62–1.21 μg/L, and 0.11 μg/L for persons entering the cabin 10 min after spraying. Overall, the potential inhalation and dermal exposures from disinsection are lower than the acceptable daily intake for d-phenothrin (ADI = 0.07 mg/kg bw).									
Berger-Priess (2004) [25]		**Objective signs of toxicity:** Calculated inhaled doses for sprayers: 3–12 μg pyrethrins; for passengers: 4–17 μg pyrethrins. Calculated dermal doses for sprayers: 200–830 μg pyrethrins per person; for passengers: 120–300 μg pyrethrins per person. Active ingredients determined on individual body parts strongly varied. For sprayers left upper arm and forearm were the most affected body parts (maximum 24 μg pyrethrins), while for passengers, it was the head and thighs (maximum 15 μg pyrethrins). **Comments:** Study personnel wore protective breathing masks and clothing; reported study was not suitable to monitor health symptoms.									
Bitelli (1969) [36]		**Subjective symptoms:** Aerosol disinsectants could cause skin irritation, irritate the mucous membranes, and, if in sufficiently high quantities, can cause systemic effects such as nausea, vomiting, fatigue, and other nervous system manifestations. **Subjective tolerability:** Passengers complain of “heavy air” and unpleasant odors, especially for longer procedures, including pre-embarkation disinsection. **Comments:** DDT mentioned but not directly linked to study personnel.									
Bonta (2003) [30]		**Subjective symptoms:** 38 self-reports consistent with exposure to pyrethroid pesticides on 237 flights, of which 95% followed residual spray applications									
Brooke (1971) [18]		**Subjective symptoms:** Acute respiratory discomfort caused by pyrethrins/Tropita1 to the authors and four engineers.									
De Tavel (1967) [37]		**Subjective symptoms:** No adverse effect on reaction or visual performance noted. **Objective signs of toxicity:** No alterations of blood cholinesterase levels noted. **Subjective tolerability:** Dichlorvos spares irritation of eyes and air passages.									
Edmundson (1970) [31]		**Objective signs of toxicity:** Participant A had little change in DDT and DDE levels; levels were comparable to white general population of the area (DDT x < 4 ppb, DDE x 9 ppb, DDA x < 2 ppb)—higher than in the general population but less than in the other participants. Participants B, C, and D showed a rise in DDT (14 ppb; 11 ppb; 24 ppb) and DDE (9 ppb; 7 ppb; 24 ppb) on the first day and then stabilized to ~4/5 ppb of their mean level in participants B and D and ~10 ppb in participant C, respectively. Statistical analyses were not presented; however, authors suggest that concentrations of DDT, DDE, and DDA were unrelated to either the amounts of aerosol used in a day or to time spent in actual spraying.									
Kilburn (2004) [28]		**Morbidity:** Five flight attendants retired due to disability. **Objective signs of toxicity:** Impaired balance, decreased grip strength in left arm, and color discrimination in both eyes; Total abnormalities: 2.8 ± 3.5 in E group vs. 1.2 ± 1.6 in NE group; *p* = 0.001. **Subjective symptoms:** Flight attendants exposed to disinsection were significantly more likely to report higher frequencies of neurological perturbation, respiratory issues, gastrointestinal discomfort, dermatological abnormalities, and sensory complaints. The Profile of Mood States (POMS) average score was also significantly higher in exposed attendants (52 vs. 21), indicating increased depression, tension, fatigue, confusion, and decreased vigor. Additionally, exposed attendants reported numb fingers (n = 18), anemia (n = 16; not quantified), sun-induced rash (n = 13), and excessive hair loss (n = 12), although no control comparison was provided. The average symptom frequency was 5.0 in exposed attendants compared to 2.6 in non-exposed attendants.									
Liljedahl (1976) [20]		**Subjective symptoms:** Irritation was not reported by study participants. **Subjective tolerability:** Odour due to disinsection was not reported by study participants.									
Maddock (1961) [26]		No subjectively reported symptoms or objective signs of toxicity were reported by study personnel.									
Przyborowski (1962) [32]		**Morbidity:** Twelve persons were hospitalized for at least a few days; 2 for 3 weeks. **Adverse event:** Seizures (n = 14). **Objective signs of toxicity:** Vitals: hypertension, bradycardia (n = 3); Labs: hypochloremia, serum bilirubin elevated or at ULN; Imaging: encephalogram (n = 1) showing signs of epileptic type; approximately 70% of samples tested were positive for dieldrin contamination. **Subjective symptoms:** Gastrointestinal: vomiting, nausea, abdominal cramps; Neurological: headache, dizziness, convulsions (involving brief loss of consciousness, frothing at the mouth, face contortions, biting of tongue and lips, and severe back spasms), falls (with loss of consciousness), dizziness, severe weakness, limb paralysis, blurred vision, tremors, isolated muscle contractions; Psychiatric: severe agitation, mania; Musculoskeletal: myalgia (n = 1); Systemic: fever (n = 2); Dermatologic: bruising and contusions associated with falls.									
Smith (1972) [27]		**Objective signs of toxicity:** A statistically significant difference in the effect of dichlorvos on plasma or erythrocyte cholinesterase activity, palmar sweating, dark adaptation, and bronchiolar resistance, between ground level, altitude without dichlorvos, and altitude with dichlorvos was not detected. No evidence that dichlorvos at exposure levels far in excess of those proposed for disinsection possesses toxicity at 8000 ft, a cabin altitude which is seldom exceeded in normal airline operations involving pressurized aircraft.									
Sutton (2007) [33]		**Objective signs of toxicity:** Specific signs of toxicity included runny nose (n = 1), wheeze (n = 1), eye conjunctivitis (n = 2), and skin erythema/flushing (n = 1). **Subjective symptoms:** The most common signs and symptoms experienced were respiratory (n = 12), nervous system (n = 11), dermatological (n = 9), eye (n = 9), cardiovascular (n = 5), and gastrointestinal (n = 6).									
Vanden Driessche (2010) [35]		**Adverse event:** Anaphylaxis. **Objective signs of toxicity:** After spraying, passenger developed facial erythema, slightly edematous eyes, pronounced lip swelling, and prolonged expiration. Blood pressure and heart rate were normal. **Subjective symptoms:** Passenger developed diarrhea and feeling of losing consciousness shortly after cabin spraying. Symptoms improved with inhaled albuterol and oral corticosteroids. Subsequent non-disinsection exposures to pyrethroid-containing compounds caused wheezing and itchy, swollen eyelid.									
Wei (2012) [29]		**Objective signs of toxicity:** Flight attendants on disinsected flights showed significantly higher levels of metabolites immediately post-flight and 24 h later, compared to pre-flight levels. Creatinine-adjusted concentrations of 3-PBA in post-flight samples ranged from 2.18 to 71.0 μg/g, decreasing to 1.20–19.2 μg/g after 24 h, while non-disinsected flights showed no significant changes. Flight attendants on disinsected flights also had higher pre-flight metabolite levels than those on non-disinsected flights. There was no significant difference between non-disinsected flights and the general population. The highest levels were found in flights to/from Australia compared to US domestic and other international flights.									
Woodyard (2001) [34]		**Morbidity:** Three flight attendants retired due to disability. **Adverse event:** blood-cell disease reported by one flight attendant. **Objective signs of toxicity:** One flight attendant reported below-normal oxygen retention. **Subjective symptoms:** Passengers, flight attendants, and pilots reported burning eyes (n = 2), severe nausea, headaches, burning skin (n = 2), itchy eyes (n = 2), loss of appetite (n = 2), acute rash (n = 2), difficulty breathing (n = 2), short-term memory loss (n = 3), difficulties concentrating, tremors, nosebleeds, long term disability (n = 3), impaired ability to fly (n = 2), congested sinuses, sore throat, difficulties swallowing, and confusion. **Subjective tolerability:** Passengers, flight attendants, and pilots complain about odour, actively try to escape disinsection.									
‡ Cawley (1974) [19]		**Subjective tolerability:** lower concentrations were less noticeable, some found odour pleasing, and S-2539 Forte was odourless									
‡ Jensen (1965) [24]		**Subjective tolerability:** None of the passengers on any flight showed awareness (viewed, heard, or smelled) that disinsection occurred.									
‡ Sullivan (1972) [23]		**Subjective tolerability:** A statistically significant passenger objection rate to higher doses of active material (1% vs. 2%) was reported (6.21 ± 7.17 and 23.26 4.39, respectively). Passenger objection to resmethrin 2% was the same as the control, suggesting 2% resmethrin was the best material tested.									
‡ Sullivan (1964) [22]		**Subjective symptoms:** Irritation from SRA aerosol was not reported by study participants, while G-1492 caused nasal dryness in a few passengers.									
‡ Sullivan (1962) [21]		**Subjective tolerability:** Unfavourable reactions to SRA aerosol were not identified, whereas G-1480 received unfavourable reactions, given a higher pyrethrum content.									

**‡:** studies reporting only subjective tolerability; **ADI:** acceptable daily intake; **con:** control group; **DDA:** dichlorodiphenylacetic acid; **DDE:** dichlorodiphenyldichloroethylene; **DDT:** dichlorodiphenyltrichloroethane; **E:** exposed to disinsection; **ft:** feet; **int:** intervention group; **mg/kg bw:** milligrams per kilogram of body weight; **ND:** no data; **NE:** not exposed to disinsection; **PBA:** 3-phenoxybenzoic acid; **ppb:** parts per billion; **SRA:** standard reference aerosol; **μg:** microgram; **ULN:** upper limit of normal.

**Table 3:** Summary of Findings: Safety, Toxicity, and Tolerability of Conveyance Disinsection.

Insecticide Compared to Control (No Insecticide) During Conveyance Disinsection Population: Humans Setting: Aircrafts Intervention: Disinsection Comparison: No Disinsection Outcome: Objective and Subjective Human Health Effects									
Stratification	No. of Studies *	Absolute Number (%)	Broad Human Health Effects (n, %)	Overall Risk of Bias	Inc.	Ind.	Imp.	Certainty of Evidence (GRADE)	References
Morbidity	3	22/62 (35.5%)	Early retirement (8/42, 19.1) Long-term disability (8/42, 19.1) Hospitalization (14/20, 70) Workdays lost (~78)	Serious	Very High	Very High	Very High	Very Low ⊕**◯◯◯**	Kilburn (2004) [28]; Przyborowski (1962) [32]; Woodyard (2001) [34]
Adverse Events	3	16/30 (53.2%)	Blood-cell disease (1/1, 100) Anaphylaxis (1/9, 11.1) Seizures (14/20, 70)	‡ N/A	N/A	N/A	N/A	‡ N/A	Przyborowski (1962) [32]; Vanden Driessche (2010) [35]; Woodyard (2001) [34]
Objective Toxicity (per physical examination and/or laboratory investigations)	9	72/105 (68.6%)	Anemia, Not Quantified (16/33, 48.5) Epileptic encephalogram (1/20, 5) Eye conjunctivitis (3/16, 18.8) Impaired cardiovascular function (3/20, 15) Impaired pulmonary function (6/25, 24) Lip edema (1/4, 25) Skin erythema (2/16, 12.5) Serum/urine insecticide metabolites detected (15/15, 100) (37–87 ppb/0.30–81.5 ppb, respectively)	Very Serious	Very High	Very High	Very High	Very Low ⊕**◯◯◯**	Edmundson (1970) [31]; Kilburn (2004) [28]; Maddock (1961) [26]; Przyborowski (1962) [32]; Smith (1972) [27]; Sutton (2007) [33]; Vanden Driessche (2010) [35]; Wei (2012) [29]; Woodyard (2001) [34]
Subjective Symptoms	8	119/123 (96.8%)	Cardiovascular (5/12, 41. 7) Dermatological (24/54, 44.4) Epistaxis (3/9, 33.3) Fever (2/20, 10) Gastrointestinal (15/51, 29.4) Hair Loss (12/33, 36.4) Musculoskeletal (1/20, 5) Neurological (54/102, 52.9) Ocular (13/21, 61.9) Respiratory (20/27, 74.1) SCIP (38/38, 100)	Very Serious	Very High	Very High	Very High	Very Low ⊕**◯◯◯**	Bonta (2003) [30]; Brooke (1971) [18]; Kilburn (2004) [28]; Maddock (1961) [26]; Przyborowski (1962) [32]; Sutton (2007) [33]; Vanden Driessche (2010) [35]; Woodyard (2001) [34]
Subjective Tolerability	1	84/591 (14.2%)	Malodour (84/591, 14.2)	Very Serious	Very High	Very High	Very High	Very Low ⊕**◯◯◯**	Sullivan (1972) [23]

* Insufficient data reported from remaining studies represented in Table 2 to be considered in calculation; ‡: Case series only, risk of bias and GRADE cannot be determined; Abbreviations: **Inc:** Inconsistency; **Ind:** Indirectness; **Imp:** Imprecision; **ppb:** part per billion; **SCIP:** Symptoms consistent with insecticide poisoning.

### 3.4. Quantitative and Qualitative Synthesis

#### Summary of Findings—Safety and Toxicity

The Summary of Findings Table 3 provides a quantitative synthesis of aircraft disinsection safety, toxicity, and tolerability. Where age and/or sex were reported [27,28,29,31,34,35], participants were mostly in young to middle adulthood, with men outnumbering women at a ratio of 1.75 to 1. Participants also largely represented those employed by the aviation industry as flight attendants, crew, pilots, technicians, or engineers [18,19,20,21,22,24,28,29,30,31,32,33,34,36], authors of the studies themselves [17,18,20,25,26], and passengers [21,22,23,24,30,34,35,36].

Among studies reporting attributable morbidity in just over one-third of exposed participants [28,32,34], notable findings included 8 individuals (19%) reporting early retirement due to symptoms related to disinsection, 8 individuals (19%) reporting long-term disability related to disinsection, 14 individuals (70%) reporting hospitalization, and an estimated 78 work days lost due to disinsection (Table 3).

Among studies reporting specific adverse events attributable to aircraft mosquito disinsection in more than 50% of exposed participants [32,34,35], notable findings included 1 case of anaphylaxis, 1 case of blood-cell disorder, and 14 cases of seizure (Table 3).

Among studies reporting objective evidence of toxicity attributable to aircraft mosquito disinsection in almost two-thirds of exposed participants [17,25,26,27,28,29,31,32,33,34,35], notable findings included detectable serum and/or urinary metabolites of insecticides (15/15 tested) [29,31]; epileptic encephalogram (5%); impaired cardiovascular function (15%); and impaired pulmonary function (24%). Additionally, objective signs of insecticide toxicity included conjunctivitis (19%); lip edema (25%); skin erythema (12.5%); and anemia (48.5%) (Table 3). One study with a comparator arm of unexposed controls [28] noted that exposed flight attendants had a significantly higher number of neurocognitive abnormalities on objective and validated testing compared to unexposed controls (mean 2.8 versus 1.2, respectively), and one cohort study of flight attendants both exposed and unexposed to disinsection demonstrated significantly elevated urinary concentrations of pyrethroid metabolites in those flying disinsected routes compared to those not [29]. Moreover, the urinary concentration of pyrethroid metabolites in the unexposed flight attendants (ie, those not flying routes that were disinsected) did not differ from that of the general population [29]. The flights conferring the highest urinary metabolites of pyrethroids in exposed flight attendants were those to and from Australia [29].

Among studies reporting subjective symptoms attributable to aircraft mosquito disinsection in 97% of exposed participants [18,20,26,28,30,32,33,34,35], notable findings included reports of systemic symptoms including fever (10%) and myalgia (5%); dermatologic symptoms such as rash (44%) and hair loss (36%); neurologic symptoms (53%) such as numbness, impaired concentration, loss of consciousness, headache, and impaired memory; respiratory symptoms (74%) such as shortness of breath; cardiovascular symptoms (42%); gastrointestinal symptoms (29%) such as nausea and diarrhea; and finally other localizing symptoms such as epistaxis (33%) and ocular symptoms (62%) (Table 3). Among 38 eligible participants in one study, 100% had symptoms consistent with insecticide poisoning [30]. In the one study with a comparator arm of unexposed controls, the mean frequency of 35 specific symptoms was almost twice that of unexposed controls [28].

Among studies reporting subjective tolerability in the absence of specific symptoms [19,20,21,23,24,34,36,37], malodour was reported by 14% of participants (Table 3).

Given the absence of studies with comparator arms across standardized methodologies, pooled summary estimates of safety and toxicity, such as relative risks and odds ratios, could not be calculated.

### 3.5. Health Equity and Human Rights Considerations

The body of main and supporting literature related to human safety and toxicity of disinsection reported very few of the PROGRESS-CANDALS factors and did not stratify the occurrence of specific outcomes according to such factors. In general, only a handful of studies or reports investigating the human health effects of disinsection, including safety, toxicity, and tolerability, reported the sex [27,31,34,35] and/or age [27,28,29,31,35] breakdowns of their participants. Given that women are more likely to be employed as flight attendants, studies of flight attendants in particular skewed more to female participants. A greater number of studies or reports of human health effects noted participants’ occupations as flight attendants, pilots, and crew [19,20,21,22,24,28,29,30,32,33,34,36] or technicians and engineers [18,31], as such employment engendered occupational exposure to disinsection. However, no studies stratified their findings according to PROGRESS-CANDALS factors as none were designed a priori or powered for such subgroup analyses.

## 4. Discussion

The systematic review identified that high-quality studies evaluating the safety, toxicity, and tolerability of aircraft disinsection were unidentifiable through our search strategy, and if such data exist, they are absent from the published medical literature and grey literature that is publicly available. Moreover, reports of health equity and human rights considerations of disinsection were absent from the included literature as well. Given that women of childbearing age are over-represented amongst cohorts of flight attendants, the reporting of health and safety outcomes according to sex and pregnancy status is of paramount importance. Of particular concern is the breadth and quality of evidence surrounding human health impacts, as no high-quality studies investigating the safety, toxicity, or tolerability of disinsection were identified. Rather, the literature base describing human health effects of disinsection comprises very limited post hoc public health surveillance, small cohort studies, one unmatched case–control study, case series, and case reports. Despite this, multiple countries have national-level regulations regarding aircraft and marine vessel disinsection using aerosolized and liquid formulations of insecticides such as permethrin and phenothrin (Supplementary Tables S2 and S3).

Experimental trials that commented on safety and tolerability in humans were primarily designed to test the insecticidal efficacy of disinsection, and standard human subject considerations and measures of methodological rigor in clinical research were largely ignored or not reported. The highest quality cohort study (which reasonably adhered to acceptable standards of methodological rigor) of flight attendants noted demonstrable elevations of urinary pyrethroid metabolites in those flying disinsected routes compared to those who did not. That particular study did not extend its findings to either subjective or objective measures of human toxicity; as such, the implications of elevated urinary pyrethroid concentrations in this context remain unknown. The scant safety and toxicity literature base has a high risk of bias; however, given the reports of significant morbidity, adverse events, and toxicity putatively and objectively attributable to aircraft disinsection, well-designed clinical and occupational health studies investigating the full range of human health impacts of disinsection on passengers and crew are urgently needed.

Current guidance on methodological components to include in studies of disinsection efficacy [16] are process process-oriented, and we could identify no corollary guidelines for assessing the safety and toxicity of aircraft or other conveyance disinsection. The long history of insecticide approval for use aboard aircraft and in many other occupational and household contexts is almost certainly underpinned by *human* safety and toxicity data that were unavailable publicly and unidentifiable by our search strategy. Making such reports available not just to regulatory agencies responsible for adjudicating safety, but to all stakeholders, including the public, in a comprehensive and organized manner, is a matter of urgency.

Based on the types and quality of safety and toxicity reports included, the human health effects of aircraft disinsection procedures are unknown. What literature does exist is concerning for a potential safety signal and, as a matter of urgency, is establishing safety and toxicity specifically of aircraft and marine vessel disinsection through methodologically rigorous, prospective, registered, IRB-approved, sufficiently powered, placebo-controlled (i.e., sham disinsection) human trials with adequate post-exposure follow-up. Reported outcomes in such trials should focus on the safety and toxicity of insecticides across the human lifespan and across the range of exposures one would expect to encounter both occupationally and as a passenger, and should include both subjective and objective measures of safety. Furthermore, a specific focus of safety and toxicity outcomes in the most vulnerable of potentially exposed individuals—including children, pregnant women and women of childbearing age, and those marginalized according to PROGRESS+ factors—should be prioritized, and studies should be sufficiently powered to report their outcomes accordingly.

## 5. Conclusions

The full spectrum of human-based outcomes related to aircraft disinsection remains incompletely understood, and accrual of primary data through high-quality research is an urgent research agenda. Additionally, there is a severe paucity of synthesized information regarding the safety and toxicity of marine, rail, and land conveyance disinsection, which are also utilized in national and regional-level vector-management systems globally. Given the potential hazards associated with insecticide exposure, particularly among those most vulnerable, such as pregnant women and children, capturing the full range of human health effects of disinsection procedures is critically important.

Current literature is limited almost entirely to small cohort studies, unmatched case–control studies, case series, and case reports, with an absence of standardized methodology, comparator groups, and ethics or institutional review board approval. As such, the human health effects of aircraft disinsection procedures remain unknown, acknowledging the inherent limitations of this systematic review given the quality and design of available studies. To address these limitations, methodologically rigorous, prospective, registered, IRB-approved, and sufficiently powered trials with both subjective and objective measures of safety and toxicity, and adequate post-exposure follow-up, are urgently needed. Comparator trials employing sham disinsection versus standard operating procedures should be pursued. Future research should also prioritize vulnerable populations, including children, pregnant women, and those marginalized according to PROGRESS+ factors, to ensure that safety and toxicity outcomes are representative and equitable.

## Figures and Tables

**Table 1:** PICO (population, intervention, comparator, outcome framework) for the systematic review of disinsection safety, toxicity, and tolerability.

Question: What is the human safety, toxicity, and tolerability of international travel conveyance disinsection versus no disinsection of aircraft, marine vessels, rail and land vehicles, to prevent or reduce the dissemination of mosquitoes through international travel?	
**Population**	Travelers and crew on any conveyance (air, marine, or land)
**Intervention**	Disinsection of all areas of any international conveyance (aircraft, marine vessels, ground and rail transport) (By specific insecticide [5] or non-chemical agent, method of application, and other).
**Comparator**	No disinsection of any areas of international conveyances (aircraft, marine vessels, and ground or rail transport)
**Outcome**	(i) Unintended consequences: (a) to individual health (travellers and staff); (b) to health equity and human rights with special attention to children and people with chronic condition such as asthma and according to the PROGRESS-CANDALS framework.

## Data Availability

All available data are contained in the manuscript.

## Supplementary Materials

The following supporting information can be downloaded at: https://www.mdpi.com/article/10.3390/toxics13110965/s1, Table S1: WHO-recommended aircraft cabin disinsection procedures, Table S2: Countries and territories with Aircraft Disinsection Regulations, Table S3: Disinsection Regulations for Marine/Submarine Conveyances. References [38,39,40,41,42,43,44] are included in the Supplementary Material.
