# Supplementary material for: A Systematic Review of Aircraft Disinsection Safety, Toxicity, and Tolerability
Source: Toxics. 2025 Nov 9;13(11):965. doi: 10.3390/toxics13110965 (PMC12656498; doi:10.3390/toxics13110965)
Supplement: Supplementary file 1 [file toxics-13-00965-s001.zip › toxics-3917666-supplementary.pdf]

Supplementary Table S1. WHO-recommended aircraft cabin disinsection procedures

Methods	Insecticide	Applied by whom	Applied when	Additional comments	Air conditioning
**Pre-embarkation cabin treatment**	Permethrin 2% aerosol at a rate of 35 g / 100 m^3	Ground staff.	Before embarkation of passengers, at the departing airport.	Should be performed in conjunction with cargo hold disinsection if cargo holds were not previously treated with residual spray.	Must be turned off during application of spray and for 5 min after completion of spraying. Recirculation fans may be left on if essential for aircraft operation, but at lowest flow rate.
**Pre-departure method**	Aerosol of d-phenothrin 2% or 1R-trans-phenothrin 2% at a rate of 35 g / 100 m^3	Crew members.	After passenger embarkation but before the overhead lockers are closed and the aircraft is pushed back for departure.	Should be performed in conjunction with cargo hold disinsection if cargo holds were not previously treated with residual spray. All areas of the aircraft cabin are sprayed, including flight deck, open overhead and coat lockers and toilets.	During disinsection and for 5 min after completion of spraying, the aircraft's air-conditioning should be set off or to normal flow, and recirculation fans must be on.
**Pre-departure cargo hold disinsection**	Single-shot aerosol can with a vertical ejection nozzle containing permethrin 2% and d-phenothrin 2% (or 1R-trans-phenothrin 2%) or an aerosol containing d-phenothrin 2% or 1R-trans-phenothrin 2% at a rate	Ground staff.	Occurs at last departure airport after all cargo has been loaded and just before cargo hold door is closed. If small animals are to be loaded, should occur before animals are loaded but after all other cargo is on board.	Only applies if holds were not previously treated with residual spray.	Must remain off during disinsection and for 5 min after completion of spraying; recirculation fans may be left on if essential but should be set to the lowest rate

	of 35 g / 100 m^3				
**On-arrival disinsection method**	Aerosol of d-phenothrin 2% or 1R-trans-phenothrin 2% at a rate of 35 g / 100 m^3	Crew members.	On arrival.	To be conducted if airline has not conducted one of the approved pre-arrival procedures, authorities at arrival airport are not satisfied that operator has used the chosen method correctly, or additional on-arrival treatment is required by authorities at arrival airport. All galleys, toilets, lockers, crew rest areas, and flight deck to be sprayed with a 5-min saturation period to be observed.	Must remain off during disinsection and for 5 min after completion of spraying; recirculation fans may be left on if essential but should be set to the lowest rate
**Residual treatment**	Permethrin 2% EC at a rate of 0.2 g a.i./m^2 for internal surfaces, 0.5 g a.i./m^2 on floors	Trained, profession al pest-control operators.	Every 8 weeks, when there are no passengers on board.	Residual treatment of the cabin should be performed in conjunction with cargo hold disinsection.	Must be turned off during treatment, but system and recirculation fans must be reactivated and run for at least 1 hour or in accordance with a.i. label guidance before passengers can embark the aircraft.

Supplementary Table S2. Countries and territories with Aircraft Disinsection Regulations [1-2]

Country	Regulation
United States of America (Arrival) Guidelines for Disinsection 2021: https://www.transportation.gov/airconsumer/spray	
Ecuador*	Countries requiring the disinsection of all in-bound flights with an aerosolized spray while passengers are on board
Grenada	
Guyana	
India	
Kiribati	
Madagascar	
Panama	
Seychelles	
Unitedd Republic of Tanzania (the)	
Timor-Leste	
Trinidad and Tobago	
Uruguay	
Zimbabwe	
Australia	Countries requiring the disinsection of all in-bound flights but allowing the residual method of disinsection
Barbados	
Chile	
Cook Islands	
Fiji	
Jamaica	
New Zealand	
Czech Republic	Areas of contagious diseases
Egypt	Zika-affected countries
France	Areas of malaria, yellow fever and dengue fever
China, Hong Kong SAR	All incoming aircraft from Zika-affected countries designated as WHO Category 1 or Category 2
Indonesia	Areas affected by any sort of infectious or contagious disease**
Italy	All aircraft coming from areas affected by Zika virus transmission and areas where the *Aedes aegypti* carrier is present

Mauritius	Flights from African continent, Asia and sub regions, the Middle East and islands of the Indian Ocean, and any other country where mosquito borne diseases are prevalent
Macau	Flights from areas of major infectious disease or Zika-affected countries
Palau	Non-US carriers from Korea, Hong Kong, Macau and Thailand
Peru	Some in-country flights
Republic of Korea (the)	30 countries, not including the United States
South Africa	Areas of malaria or yellow fever
Switzerland	Intertropical Africa
Taiwan, China	Incoming flights from areas with arbovirus vectors *Aedes aegypti* and *Ae. albopictus*
Thailand	Areas of yellow fever
United Kingdom of Great Britain and Northern Ireland	Malarial countries and countries with confirmed transmission of Zika (Voluntary)
Canada (Arrival) Guidelines for Disinsection ~2017: https://www.aircanada.com/ca/en/aco/home/plan/peace-of-mind/travel-tips.html#/	
Australia	Pre-embarkation Method (Primary Method) – This method takes place without passengers or crew on board, and is performed or supervised by a Certificate holder.<br><br>On Arrival Method (Alternate Method) – This method takes place before passengers have disembarked and the doors have been opened. Crew walk through the cabins discharging approved single-shot aerosols in the prescribed dosage.
New Zealand	
Aruba	Top of descent method: This method is similar to the "On arrival method", except that it is carried out at the top of the aircraft's descent, just before it starts preparations for landing.
Barbados	
Buenos Aires (Argentina)[3]	
Cuba	
Guadeloupe	
Jamaica	
Martinique	
Puerto Rico	
St-Lucia[1]	

St. Vincent and the Grenadines
Santiago (Chile)[2]
Trinidad and Tobago
Turks & Caicos

* only Galapagos and Interislands

**Indonesia is sensitive to infectious diseases due to population health and climate which helps propagate infectious disease such as SARS and influenza from China, MERS CoV from the Middle East, and HIV from areas in Africa. [3]

[1] Spraying only required if flight transits at another destination before arrival.

[2] Spraying is required YYZ - SCL before landing at Santiago, Chile, and SCL - EZE before the aircraft arrives at Buenos Aires.

[3] Spraying is required EZE - SCL before arriving in Santiago, Chile.

Supplementary Table S3. Disinsection Regulations for Marine/Submarine Conveyances

General Ship Regulations		
All vessels must obtain a Ship Sanitation Control Certificate or a Ship Sanitation Control Exception Certificate every six months, as stated by the International Heath Regulations. This certificate should include the presence of any pest or insect as well as the method used to eradicate them from the vessel. If the certificate has expired or they do not have one, the ship will undergo inspection and further disinsection in places specifically where vectors (such as mosquitoes, cockroaches, etc.) fester as outlined by the WHO [4].		
**Specific regulations**		
Conveyance type	Country	Regulation
Ship	China	Yellow fever prevalent area including Nigeria, Peru, and Brazil ((except for Ceará, North Rio Grande, Texas, Paraiba, Pernambuco, Alagoas, Sergipe) [5].
	Republic of Korea	Zika affected countries; Certificate must indicate used of pyrethroid ingredient in insecticide used for disinsection. Disinsection must occur at least 1 hour prior to departure from affected countries [6].
	European Union (EU)*	Ports in the European Union Member States (EUMS) to disinsect the imported goods at the port facilities [7].

***EU Countries include** Austria, Belgium, Bulgaria, Croatia, Republic of Cyprus, Czech Republic, Denmark, Estonia, Finland, France, Germany, Greece, Hungary, Ireland, Italy, Latvia, Lithuania, Luxembourg, Malta, Netherlands, Poland, Portugal, Romania, Slovakia, Slovenia, Spain and Sweden.

References:

[1] *Aircraft Disinsection Requirements | US Department of Transportation*. https://www.transportation.gov/airconsumer/spray.

[2] Air Canada - health and travel tips. Air Canada. (n.d.). https://www.aircanada.com/ca/en/aco/home/plan/peace-of-mind/travel-tips.html#/

[3] Subiakto, Yuli. "Aviation Medicine Capacity on Facing Biological Threat In Indonesia Airports." *Infectious Disease Reports*, vol. 12, no. Suppl 1, July 2020, p. 8738. *PubMed Central*, https://doi.org/10.4081/idr.2020.8738.

[4] Canada, Health. *ARCHIVED - Ship Sanitation Certificate Program*. 4 Feb. 2009, https://www.canada.ca/en/health-canada/services/healthy-living/travel-health/general-advice/ship-sanitation-certificate-program-health-canada.html.

[5] *China: Yellow Fever Prevention Measures - The Swedish Club*. 12 Feb. 2018, https://www.swedishclub.com/news/loss-prevention/china-yellow-fever-prevention-measures/.

[6] *Zika Virus: South Korea Requires Self-Disinfection Certificates for Vessels - The Swedish Club*. 24 Mar. 2016, https://www.swedishclub.com/news/loss-prevention/zika-virus-south-korea-requires-self-disinfection-certificates-for-vessels/.

[7] Interim guidance on maritime transport and zika virus disease. (n.d.). https://www.shipsan.eu/Portals/0/docs/MaritimeZika_EUSHIPSAN_UPDATE_13.4.2016.pdf
